# Periscope; or, Circumspect Review

**Published:** 1828-04-01

**Authors:** 


					l82s] ( 505 )
OK,
CIRCUMSPECTIVE REVIEW.
" Ore trahit quodcunque potest, atque addit acervo."
[23d FEBRUARY, 1828.]
1- Inflammation by Contiguity. By
Drs. Graves and Stokes.
There are many reasons for believing
that inflammation is sometimes propa-
gated by the mere contact of a sound
with an inflamed part. Drs. Graves and
Stokes furnish us with the following
curious fact. In the dissection of a fatal
case of enteritis, it was observed that
the omentum, which lay extensively over
the intestines, was healthy, except where
it was in contact with the inflamed por-
tions of the latter. These portions (of
intestine) were Circumscribed and limited
in extent?some being highly vascular
and red?others gangrenous?and one
perforated. The inflamed portions of
omentum were very vascular and red,
about the size of a dollar, and lay exactly
over the inflamed portions of intestine.
Similar phenomena have been often ob-
served in other parts of the body, but no
explanation has been attempted. Thus,
when the pleura costalis is much in-
flamed, a portion of the pi. pulm. cor-
responding or opposite to it, is always
found inflamed also. So, when inflam-
mation spreads from the pleura to the
lungs, it does not follow the reflections
of this membrane, but passes directly
from the pleura of the ribs to the pleura
of the lungs, without any communication
of vessels. The following explanation
is offered by the gentlemen in question.
" When a portion of a serous membrane
becomes inflamed, it is rendered highly
vascular; it becomes at first dry and
rough, but afterwards exhales either a
morbid fluid secretion, or coagulable
lymph ; there is some reason to believe
that its temperature is also increased.
Now in this state of things, that portion
of the opposite membrane which corres-
ponds to it, is thus exposed to the contact
of a membrane, whose sensible properties
are altogether altered from their natural
state, and which may therefore be now
considered to be as it were a foreign body,
which presenting a surface quite different
from that to which the sensibility of the
opposite membx*ane had been accustomed,
must of course act as a stimulus to it,
and thereby excite in it an inflammatory
action." This explanation seems at least
as satisfactory as Mr. Hunter's " Sym-
pathy of Contiguity."
2. Encysted Dropsy op Abdomen cured
by an Operation. By Dr. Morton,
of Huntingdon.
In a late Fasciculus (II.) of this Number,
we presented our readers with a case of
hydatids of the liver cured by an ope-
ration in the Hotel Dieu. In the first
Number of our Glasgow cotemporary
(which we hail with the most friendly
greetings) is detailed a case which bears
considerable affinity to that which oc-
curred in Paris.
Case. Twelve years before the date of
report, William Cartwright, a labourer,
had been attacked with some severe
illness, followed by a great enlargement
of the abdomen, and sense of uneasiness
in the region of the liver. When seen by
Dr. Morton, he had troublesome cough,
accelerated pulse, scanty urine, costive
bowels, and difficulty of breathing in the
recumbent posture! There were tume-
faction and fluctuation of the abdomen?
and sense of weight in the epigastrium.
Various diuretic medicines were em-
ployed, but the disease advanced. An
operation was pi'oposed?acceded to?
and performed on the (ith March, 1810.
The trocar was introduced below the um-
bilicus, in the linea alba. About an ounce
Vol. VIII. No 16. 3 K
By
506 Periscope; or, Circumspective Review. [Feb. 13
of white purulent fluid passed through
the canula, and no farther discharge could
he procured. Next day, however, a por-
tion of cyst protruded, and was drawn
away piece-meal. On the 3d day, two
other pieces of cyst were extracted?and
the same occurred on the 4th and 5th
days. After the extraction of a large
portion of cyst on the sixth day, there
gushed out two quarts of a yellowish
white fluid, containing a number of hyda-
tids, which were perfectly spherical, vary-
ing from a quarter of an inch to an inch
in diameter. On the 7th and 8th days,
nearly a quart of hydatids was discharged,
one of which measured two inches in its
longest diameter. On the 9th day, a
large portion of thickened cyst was eva-
cuated, followed by four quarts of hyda-
tids. This discharge of hydatids con-
tinued for two months, amounting to
two gallons and a half. The contents of
these cysts were as limpid as water, and
no animalcula could be discovered in
them. The orifice has never been en-
tirely closed, but discharges, at intervals,
small quantities of purulent serum, with-
out any detriment to health. After a
lapse of seventeen years, the patient is
able to earn two pounds a week, as a
paver of the streets of Huntingdon.
3. Violent Abdominal Contusion.
M. Reveille Parise lately reported a case
to the Royal Academy of Medecine, which
shews what a tremendous pressure the
human frame will sometimes bear with-
out destruction. On the 20th October
last, a gentleman, of delicate constitution,
was riding ahorse that was subject to ver-
tigo?when the animal fell, and in the
endeavour to rise fell again,, with all his
weight, directly on the body of his master!
The shock was terrible, and the animal
was seen by M. Parise, to roll completely
over the unfortunate gentleman and get
up on the other side ! M. Parise ran to
his assistance, and thought him dead;
but, in a few minutes, he revived, and
spoke with a strong voice. He was
quickly conveyed home, stripped, and
carefully examined. The thorax was
found to be uninjured, as the whole weight
of the animal had been sustained by the
abdomen. There was no fracture or lux-
ation of sacrum, vertebrae, or bones of
the pelvis. There were, however, ex-
treme anguish?pallor of the whole sur-
face?cold sweat all over the body?
feeble pulse?tremor and convulsive agi-
tation of the limbs?all which symptoms
led to the apprehension of severe lesion
of soma internal organ. There were,
besides, a dull pain in the abdomen, and
constant tenesmus. An anodyne medi-
cine was exhibited, and warm embro-
cations applied to the abdomen. Lave-
ments of a soothing kind, were also
thrown up. A large quantity of blood*
was abstracted from the arm. The next
day found the patient better than could
have been expected. The anxiety was
gone?the pulse was expanded?the agi-
tation tranquillized. But there was great
tenderness, with tension of the abdomen,
hot skin, and quick pulse. Forty leeches
were applied to the abdomen, and the pa-
tient afterwards put into the warm bath.
From this time he rapidly recovered,
without one bad symptom.?Journal
Generale de Medecine.
We think the large detraction of blood
from the arm, during the state of de-
pression and prostration in which the
patient appeared, immediately after the
accident, was a hazardous practice?and
one that ought not to be imitated *
4? Hydarthrosis, or Dropsy op the
Knee-Joint.
The following case of a rare disease is
published by Dr. Vilette, in a late Num-
ber of the Revue Medicale. On the
Gth October, he was consulted by a gen-
tleman for a swelling above the left knee^
It seemed to be divided into two parts by
the rectus femoris. The inner swelling
was the largest, and also the softest.
The patella, which was nearly immoveable,
was raised from the joint. Fluctuation
was perfectly distinct in both sacs, and a
communication between them was evi-
dent. Whether the contained fluid was sy-
novia or pus, was the question. There had
been no preceding blow or other violence
?no inflammation?no pain. The swel-
lings had appeared without warning, and
gradually increased. Mercurial frictions
and various other means were employed
without any benefit?indeed the swelling
1828] M. Hufeland on Diseases of the Fcetus in Utero. 507
had very much increased, with much pain,
and on the 11th October, the capsule of
the joint was greatly distended, and the
two tumours were now nearly in one.
The patient insisted on puncture of the
swelling, notwithstanding the remon-
strances of the doctor. He threatened
to do it himself, if M. Vilette any longer
hesitated. A trocar was, therefore, in-
troduced above the patella, and on one
side. Eight ounces of pure synovia were
evacuated?the canula was withdrawn,
and a compress and bandage placed on
the part. Next day the synovial liquor
had accumulated to nearly the same ex-
tent as before. He had not so much pain
in the knee, but there was intense pain
in the hip-joint. Ice was applied. In
three days more, the fluid had increased
much, and a second introduction of the
trocar evacuated five ounces of fluid per-
fectly clear. The joint was now en-
veloped in a large sinapism. This pro-
duced such terrible inflammation and pain
that it was removed in three quarters of
an hour. In the evening, the inflam-
mation had subsided, and the sinapism
Was renewed for an hour and a half.
There was now a considerable cutaneous
inflammation established. We need not
pursue the details. The synovial effusion
did not recur, except in a trifling degree,
which was removed by successive sina-
pisms. A complete cure was effected.
A very long train of pathological re-
flexions is appended to the above history,
but we deem the fact to be more valuable
than the commentary?and the former
We present to our readers, leaving them
to draw their own inferences.
G. On Diseases of the F<etus, with
the Means of Prevention and Cuue.
By M. Hufeland.
The venerable author of this paper has
carried his philanthropy beyond the boun-
daries of this life, as commonly, but er-
roneously computed from the day of birth,
and directed his researches to the con-
nexion of the foetus with the mother?
to the influence of maternal agents on the
embryo?to the results of these agencies
?and to the means of counteracting nox-
ious by salutary agents.
Whatever may be the evils of a redus-
dant population in this country, it must
always be the business of the medical
profession to encourage the increase of
births over deaths?and, consequently,
to protect the foetus in utero, as much as
the independent being at any other epoch
of its existence. At first sight it appears
somewhat absurd, as well as impossible,
to extend the aid of medical art to the
embryo in the womb ; but this is a false
view of the case. Although the foetus
swims in a liquid, and is consequently
secured, in a great measure, from exter-
nal succussions?and although neither
nerves nor blood-vessels can be traced
from the mother into the foetal placenta;
yet, it is evident that the materials of
organization in the little parasite must
be derived from the nidus in which it
resides, while facts are not wanting to
prove that nervous agency or influence
may be conducted from the mother to
the child, whatever may be the medium
or the mode in which it is conducted.
Thus, sudden frights have destroyed in-
stantaneously the life of the intra-uterine
foetus. Some experiments made on im-
pregnated animals prove that substances
introduced into the blood of the mother
will find its way into the veins of the
fetus. Thus, oil was injected into the
veins of a bitch with whelp, and, after a
certain time, the animal was killed, when
oil was detected in the umbilical veins
of the foetus. When the foetus is de-
tached, and put to thebreast of the mother,
we find that medicinal substances will
act through the medium of the milk, with
nearly as much certainty as if given di-
rectly to the infant. The itch, in a child
at the breast, has been cured by sulphur
exhibited to the mother. Hufeland main-
tains, that nervous and moral affections
may be readily impressed on the suckling
through the medium of the nurse. We
cannot deny that the nutrition of the foetus
is equally derived from maternal sources,
while intra uterum, and during lactation.
That the agency of heat, cold, elec-
tricity, magnetism, &c. may be extended
from mother to offspring is undeniable.
M. Hufeland thinks that metastasis also
may play some part in affections of the
foetus in utero. There can, at least, be
no doubt that the syphilitic virus may be
communicated to the germ before it has
assumed an independent existence.
From these and many other consider-
3 K2
508 Periscope ; or, Circumspective Review. [Feb. 33
ations, M. Hufeland thinks we are autho-
rised to infer, that we can act on the
foetus in utero in seven different ways
namely, by the augmentation or diminu-
tion of nourishment?by the augmenta-
tion or diminution of the afflux of blood
to the uterine system?by changing the
qualities of the air and aliment of the
mother?by mechanical means?by the
agency of electricity, &c.?by medicines
?and, lastly, by moral influences.
Let us now pass on to the diseases of
the foetal life. The first class compre-
hends monstrosities, deformities, &c.
which our author thinks must proceed
either from hereditary disposition or some
defect in the primary development of
the foetus, through causes acting On the
mother. This, however, is a knotty point
to unravel. In the second class, he ranks
the feeble state of vital powers, the effect
of defective nutrition, and the atrophy of
infants when first born. We see infants
come into the world extremely small,
feeble, and emaciated. The causes are
to be sought in diseases of the mother,
as immoderate evacuations, fevers, acci-
dents, want, anxiety of mind, &c. In
the third class he places hypertrophy, or
excess of nutrition in the foetus, whether
of certain parts, or of the whole, thus
rendering the birth difficult or dangerous.
The fourth class comprehends the dys-
crasiae. M. Hufeland contends that, as
the pabulum of nutrition in the foetus
must be supplied by the mother, so all
vices in the blood, the secretions, and
the fluids generally, must affect, more ox-
less, the foetus in utero. Hence we see
scrophulous mothers bring forth scrophu-
lous children, See. There is little doubt
now entertained, that the syphilitic virus
can be communicated to the intra-uterine
progeny?and if this be the case, we can-
not wonder that other maladies should be
also transmitted. But not only are dis-
eases thus communicated from mother to
child, but death itself may be induced in
the latter from morbid impressions on
the former. A fright?a strong moral
affliction, and many other influences, have
been followed quickly by cessation of all
motion in the foetus, and ultimate abortion.
A contemplation of these causes of de-
triment, disease, or destruction of the
child, will very readily suggest the pre-
vention, as well as the remedy?where a
remedy can be applied. The whole se-
cret, in fact, rests upon abstracting all
causes of ill health from the mother?
or, if ill health has been actually induced,
to restore her, as soon as possible, to
health. It would be well for mothers and
accoucheurs to reflect on these things,
and preserve, if possible, the innocent
foetus from the effects of irregularities
and intemperance too often indulged in,
from a false supposition among nurses,
that pregnant females cannot live too
generously.?Journ. Complement.
6. Bony Tumour obstructing thk
Pylorus.
This is a very rare disease. Dr. Webster
has lately recorded an example of it. The
patient was an elderly gentleman who
had enjoyed good health, with the excep-
tion of some attacks of dyspepsia, atten-
ded with costiveness, and relieved by
diarrhoea. These attacks never assumed
a formidable character till last Autumn,
when he was, one morning, seized with
excruciating pain in the epigastrium, ac-
companied by slight sickness and accele-
ration of pulse, anxiety, constipation, &c.
These symptoms increased in urgency,
and a fluctuation and fulness could be felt
at the epigastrium, by Mr. Nicholson,
who had been called to the patient.?
Bleeding, cathartics, enemata, all failed,
and the patient sunk in 22 hours from the
commencement of the attack.
Dissection. The stomach was found to
be much distended, and a cartilaginous
body, intermixed with numerous spiculas
of bone, was discovered firmly attached
to the coats of the organ, close to the
pylorus, into which one end of the tu-
mour projected like a stopper, thereby
preventing all egress from the stomach
into the duodenum. There were some
marks of inflammation in the stomach,
and a considerable effusion of serum into
the cavity of the abdomen.
7. Ascites cured by Injection op
Wine-vapour.
At a late sitting of the Academy of Mede-
cine in Park, M. L'Homme related a case
of ascites cured by the introduction of
1B28] Parujria Erratica. 509
the vapour of wine into the abdomen,
after the water had been drawn off. The
patient was 49 years of age, and had en-
joyed good health up to the age of thirty-
eight years, at which period he had an at-
tack of haematemesis, which lasted four
days. After this attack, the abdomen be-
gan to swell. Various means were em-
ployed, without effect, and in six months it
Was necessary to perform paracentesis.
Sixteen pints of clear water were drawn off
In one year more the operation was again
necessary ; and by the end of 1S22, when
the patient came under M. L'Homme's
care, the paracentesis had been six times
performed. The reporter tried various
diuretics, but without making any im-
pression on the dropsy. At this time he
read an account, in Broussais's Journal,
of a cure by the introduction of wine-
vapour into the peritoneal cavity, after
the evacuation of a fluid, and he deter-
mined to try this measure. After the
operation of paracentesis abdominis,
therefore, he contrived, by means of an
apparatus which cooled the vapour to the
animal temperature, to introduce the
wine-vapour six times into the peritoneal
cavity. The patient experienced no other
sensation than that of mere distention,
after theseoperations. He was prepared
to remove the vapour, if it occasioned
much inconvenience ; but this was not
the case. Only some slight colicky pains
ensued, which recurved, from time to
time, for two months. The dropsical
effusion never returned ; and it is now
two years since the operation.
M. L'Homme tried the same experiment
on another patient, who had been drop-
sical for more than 20 years ; but it failed
of success. No bad accident, however,
resulted from the introduction of the
vinous vapour.
The operator exhibited to the Academy
an apparatus which he had constructed
for this operation, and by which the
vapour raised from the boiling wine was
cooled, in its passage, to the peritoneal
cavity.?Revue Med.
8. Paruria Erratica, op Uroplania.
There is probably not a more remarkable
instance of the freaks of Nature on re-
cord, than the following case, which
appears to be well authenticated. It is
published by Dr. Arnold, in the first
Number of the American Journal of the
Medical Sciences, for November last.
Maria Brenton, aged 27 years, of sound
constitution, had enjoyed good health till
June 1820, when the catamenia became
suppressed, and hasmoptysis ensued. For
this she was bled profusely, by some irre-
gular practitioners to whom she applied,
till her system was greatly debilitated,
and then emetics were administered.
Prolapsus uteri now took place, and the
unfortunate young woman was rendered
unable to pass her urine without the aid
of a catheter. In this state she continued
for two years. In September, 1822, Dr.
Arnold first saw the patient. No water
had been drawn off for '/2 hours, and,
strange to say, " the urine found an out-
let by the right ear, oozing drop by drop,
and this continued for several hours after
the bladder had been emptied." At five
o'clock the next day, the discharge from
the ear recommenced, and a larger quan-
tity than before was poured out. This
occurrence took place for several days in
succession. The fluid had all the charac-
ters of urine. Sometimes it came out oj
the ear in a stream as large as a crow-quill,
so that a pint was discharged in the space
of 15 minutes ! This issue from the ear
was accompanied by pain about the eye
and ear of that side, and distressing
sense of fulness?especially if the fluid
did not come at the usual time, in which
case she sometimes became delirious,
convulsed, or insensible. The hearing of
that ear was lost, and also the sight of
the corx-esponding eye. After a time
there occurred a discharge from the other
ear, of a similar kind?and, some time
afterwards, the urinous fluid made its way
from the left eye, accompanied by great
inflammation in that organ. This vicar
rious outlet only continued three daysm
but a sufficient quantity of fluid was col-
lected to be tested, and proved to be of
the same composition as that which issued
from the ears. In the Summer of 1824,
this urino-lachrymation recommenced,
and continued for six weeks. Previously
to this, however, the patient had dis-
charged large quantities of the urinous
fluid from her stomach?then from the
right breast?and subsequently from the
left. After suffering some severe cramps
and pains in her abdomen, the urine
510 Periscope; or, Circumspective Review. [Feb. 23
burst forth, one day, from tho navel?
and this discharge has since continued,
with some interruptions. " Nature,
wearied in her irregularities, made her
last effort, which completed the pheno-
mena of this case, and established a dis-
charge of urine from the nose." Professor
Silliman analyzed specimens of all these
discharges, and found them to contain
urea, alkaline sulphates, muriates, phos-
phates, &c. During all this time, urine
also came from the bladder, generally of
a very high colour when first made, and
afterwards turning black. There has
been a pretty regular vicarious discharge
of blood from the stomach or lungs, ever
since the suppression of the catamenia.
Dr. Arnold feared that imposture might
be suspected, if not practised in this ex-
traordinary case, and therefore, to remove
every doubt, Dr. Webb and himself kept
watch alternately, for 24 hours, on the
patient, without, for a moment, losing
sight of the parts from which the fluids
issued. The quantity and quality of the
discharges did not vary from those ob-
served when no watch was kept. It was
very curious, (if a fact1) that, when the
urine was not drawn off from the bladder,
for 48 hours or more, the quantity found
in it was always less than when drawn off
every twenty-four hours. " Sometimes
when the urine was not drawn off for
seventy-two hours, it would amount to
only one or two ounces." In such cases,
the vicarious discharges were always in-
creased. Dr. H. allowed the bladder to
remain undisturbed for seven days at one
period, at the expiration of which, only
three ounces were found in that recep-
tacle. Her general health, mean time,
did not suffer. When, on the other hand,
the urine was drawn off every two or
three hours, the sum total of the other
vicarious discharges was greatly dimi-
nished. When the account breaks off,
" the discharges from the right ear, the
right breast and navel continue daily, but
they are not so great nor so frequent as
they were a year since. From the blad-
der the quantity is as usual?from the
stomach, nose, eye, there has, for some
months, been no discharge." A table is
appended, occupying ten pages of letter-
press, exhibiting the quantities of urinous
fluid daily discharged, from the various
outlets above-mentioned, between the
21st September, 1822, and the 30th July,
1824. This diary was kept by Messrs;
Goodwyn, and Joseph Tearing, medical
students, assisted by Mr. Greene, another
medical student.
It would be difficult to produce a ease
better authenticated than the above?and
still more difficult to adduce a train of
phenomena so hard to believe. There
are one or two phenomena, which we
cannot prevail on ourselves to admit,
without the aid of deception on the part
of the patient. One is, the urinous dis-
charge from the ear in a stream, and to
the amount of a pint in fifteen minutes.
We have seen a man smoking his pipe,
and puffing the fumes through his ears??
sometimes through one ear?sometimes
through both. Now it is not quite impos-
sible that what was done with an aeriform
fluid, might be done with a watery one?we
mean the forcing it through the eustachian
tube,and through an aperture in themem-
brana tympani. The quantity discharged
from the eye was so small, that a strong
lachrymation might give it origin. The
quantity which issued from the ear was
such, that all idea of its secretion there
must prove revolting to credibility. The
secretion from the glands of the mamma,
or even from the extensive membrane of
the nose, is not beyond the boundary of
belief, knowing, as we do, that Nature
performs astonishing freaks sometimes,
especially when interrupted in her usual
course, Neither do we hold it impos-
sible that a vicarious secretion of a uri-
nous quality, should issue from the sto-
mach, notwithstanding the obloquy which
was thrown, a few years ago, on Dr.
Yeats, in a well-known case in this
country. But what shall we say to the
urinous eruption from the umbilicus ?
Upon the whole, we conceive that the
respectable Editors of the Journal in
question, are bound to investigate the
accuracy of the report, and verify or re-
fute the statements which have been
made.
9. Typhus Fever.*
Dr. Clanny is not unknown to the medi-
* A Lecture upon Typhus Fever, as lately
delivered at the Sunderland Infirmary. By
William Reid Clanny, M.U. F.R.S. Ed.
&c. &c. Quarto, pp. 28. Longman's, Feb.
1828.
1828] Laryngotomy. 511
cal world as a sincere lover of his pro-
fession . More than twenty years ago he
published a neat analysis of the Butterby
Waters, near Durham ; subsequently an
interesting one of those of Gilsland, in-
terspersed with practical observations;
recently we had occasion to commend his
construction of an instrument, termed
the zopuron; and we have now to call
attention to his lecture on typhus fever.
During a professional career of twenty-
four years, he has especially devoted his
nrind, for the last ten, to an "inquiry
into the proximate cause, and appropriate
method of cure, of typhus j" and has con-
trived, with his usual ingenuity, a well
adapted apparatus for the chemical ana-
lysis of the venous blood of typhus pa-
tients. He has printed several tables of
the results of that analysis, which was
performed on the sixth, twelfth, and
eighteenth days of the fever, and con-
trasted them with the component parts of
the blood in a state of health. For these
tables we must refer to the lecture itself,
as well as for remarks elucidatory of them.
The following extract exhibits a brief
summary of the opinio as of the lecturer.
" In the progress of typhus fever, we ob-
serve a direct approximation in the propor-
tionals of the blood to the lymph, which cir-
culates in the lymphatic system, and nothing
but a total cessation of sanguification could
work this astonishing change in the blood,
whilst Nature, ever true to herself, causes
an increased absorption of lymph by the open
mouths of the lymphatics, from all parts of
the body, to supply the place of the chyle,
which is, as I have demonstrated, no longer
taken up from the food in the alimentary
canal, as in a state of health. This accounts
for typhus blood in advanced cases, having
only jgjg of albumen, instead of T^, as in a
state of health. The fibrin is also decreased
from to ?fife. All medical history informs
us, that the blood of typhus patients de-
creases in quantity, in a gradual manner,
from the commencement of the disease to
the turn, in favourable cases, or to a fatal ter-
mination in unfavourable cases. From these
facts I have come to the conclusion, that the
proximate cause of typhus fever is a cessa-
tion of chylification, and consequently of
sanguification, during which time the lym-
phatics of the whole system act with in-
creased vigour, and in this manner the lymph
taken up by them from the system supplies,
for the time being, the place of the chyle in
the blood, and as long as this state continues,
the patient labours under an acute disease,
heretofore called typhus fever. 'When the
chylopoietic viscera resume their functions,
the disease gradually recedes, and health is
ultimately restored. From the above facts
every symptom and phenomenon of the dis-
ease receive a ready explanation." " During
the fatal progress of the disease, carbonic
acid is not to be found in the blood, and
except a turn take place, by which fresh chyle
is carried to the thoracic duct, the blood is
rendered vapid, and, in some cases, it passes
into a putrid state."
The author assures us he has " experi-
mented upon the blood taken from pex--
sons labouring under acute diseases, and
could, in no instance, find those changes
which invariably present themselves in
typhus." His treatment consists of oc-
casional venesection ; leeches, cupping,
and blisters, for local pain, or determina-
tions of blood to particular organs ; car-
bonic acid water, or effervescing draughts,
with enemata of carbonic acid in an un-
mixed state ; and, in cases of extreme ex-
haustion, ablutions of tepid sherry wine.
Aperient medicines should be given ac-
cording to circumstances, and not in a
general way, and uniform attention to
moral causes, ventilation, and cleanliness
is indispensable.
Not having seen the apparatus of Dr.
Clanny, not having scrutinized into the
accuracy of his analyses, nor having sub-
jected either his opinions or mode of cure
to the test of experience, we can only
recommend them to the candid consider-
ation of our readers, as emanating from
an ingenious and experienced physician,
who laudably occupies his time, directs
his talents, and spends his money, for
the relief of suffering humanity, and the
advancement of his profession.
10. Laryngotomy.
Dr. Smith, the editor of the Philadelphia
Monthly Journal, has published a melan-
choly case of a girl, ten years of age, who
was attacked with severe cough and diffi-
culty of breathing soon after an attempt to
swallow a triangular piece of dried gourd-
shell. The symptoms had continued five
weeks, when Drs. Smith and M'Clelland
saw the patient. The cough now came
on in convulsive paroxysms, which pro-
duced the appearance of a sudden emphy-
512 Periscope; or, Circumspective Review. [Feb. 23
sema in the throat during each attack.
Respiration was very difficult and stridu-
lus, and the poor girl could not lie down
in bed at all. Deglutition was also very
difficult. There was some fever.
Dr. M'Clelland conceiving that a fo-
reign body was lodged within the larynx
or trachea, determined on an operation.
" An incision was commenced at the
lower edge of the thyroid cartilage by a
bold plunge with a sharp-pointed scalpel,
and carried downwards into the cavity of
the trachea, below the cricoid cartilage.
An aperture was then made about an inch
in length, through which the air instan-
taneously rushed." There was very little
haemorrhage. The symptoms were not
relieved, and therefore a long and slen-
der pair of forceps was carried down to
the bifurcation of the trachea?but no
foreign body could be found. The case
was necessarily left to the efforts of Na-
ture. After the operation, a more copious
expectoration came on, and the cough
was much relieved. The emphysematous
appearance of the neck also subsided.
The febrile symptoms continued, and the
little patient died in eight days after the
operation. On dissection, the piece of
gourd-shell was found impacted in the
left bronchus, about half an inch from
the bifurcation, and surrounded by tough
coagulated lymph. It was swollen very
much from the moisture and heat of the
parts, so that it completely obstructed
the bronchus in which it lay. There was
complete pericarditis, with several adhe-
sions of pericardium to the heart. The
left lung, as might be expected, was con-
gested, and, in a great measure, hepa-
tized, from pulmonic inflammation.
Dr. Smith considers the mode of ope-
x-ating adopted by Dr. M'Clelland as
a great improvement in tracheotomy.
Nothing," says he, " can look more
ridiculous than to see a surgeon making
a long and tedious dissection, for the
purpose of getting into so superficial a
cavity as the trachea. A bold plunge of
the scalpel accomplishes the thing at
once." Dr. S. avers that there can be
no danger of haemorrhage. In this we
cannot quite agree with him. We have
seen great haemorrhage, from a alight
wound of the thyroid gland, which pours
out blood like a large vessel. Now, by rer
peatedcuts, we do not divide many vessels
at one time, and we can tie as we go on,
if necessary; but the " bold plunge" may
throw more work on our hands, in this
operation, than may prove quite conve-
nient. High up in the throat, near the
thyroid and cricoid cartilages, we may
wound the gland, which is sometimes
irregular in its size and situation. Low
down in the neck, the trachea recedes
considerably from the surface, and a
plunge into the trachea there might be
more bold than prudent.
TRANSACTIONS of societies.
1. H/EMATEMESIS?MeLjENA.
In a late sitting of the Westminster Me-
dical Society, Mr, North furnished some
cases of the above disease, as a subject
for discussion. The first case was that
of a young woman, who had been in ap-
parent good health, up to the occurrence
of the accident, with the exception of a
suppression of the menstrual discharge,
of a few months' duration. As the vo-
miting of blood was considered to be vi-
carious of this suppression; as each dis-
charge seemed to relieve the symptoms
of oppression about the praecordia ; and,
as the patient appeared to bear up well,
both mentally and corporeally, against
thg attack, no apprehensions were enter-
tained by the medical attendants, and a
favourable prognosis was given. The
patient, however, died suddenly, a few
hours after this judgment was delivered.
Some dark grumous blood, or secretion
of a melsenic character, had been dis-
charged by stool. On dissection, the
mucous membrane of the stomach and of
the upper intestines, was found in a sof-
tened state, so that it could be easily
spraped off by the scalpel. Beneath,
there were numerous points of redness,
or of ecchymosis, both in the stomach and
bowels. No disease could be recognized
in any of the collatitious viscera.
In another case, the symptoms were so
nearly similar, that Mr. North did not
detail them. Two physicians pronounced
1828] ILematemesis?Mel^ena. 513
the disease devoid of danger?but Mr.
North, remembering the foregoing case,
gave a doubtful prognosis, notwithstan-
ding that a fellow of the college was
against him. The patient died almost
immediately after the doctors had decided
that she was not to die?at that time !
The third case was that of an old woman,
and consequently the gastrorhagia was
not vicarious. No dissection was per-
mitted. Mr. North remarked that, in his
researches among authors on the subject
of hsematemesis, they appeared to him to
consider a discharge of blood from the
stomach, when vicarious, and when it was
not accompanied by severe or threaten-
ing symptoms, as always devoid of dan-
ger. It was upon this principle that the
confident and favourable prognosis was
given in the first two cases by the phy-
sicians. It was argued, however, by Dr.
Johnson, Dr. Barry, and Dr. Copland,
that the disease should not be held so
cheap?for, in the first place, it might
not always be safe to pronounce a vomit-
ing of blood vicarious, because a suppres-
sion of the menstrual or hsemorrhoidal
flux had preceded the accident?and, in
the second place, the hsematemesis is so
very frequently the effect of disease in
some of the collatitious viscera, as the
liver, spleen, &c. that it maybe, and too
often is, attended with considei'able dan-
ger. Dr. Johnson, in particular, con-
tended that the disease could not be pro-
nounced devoid of danger, unless it was
periodical, and unequivocally vicarious
of some other discharge. When it hap-
pened, for the first time, no man could
tell what might be the event. He ob-
served that a simple and accidental vo-
miting of blood, unattended with any
melaenic discharge from the bowels, was
infinitely less dangerous than when a
black secretion, like tar, constituting
melama, issued per anum. This last af-
fection is always a dangerous disease.
Dr. Barry did not agree with Mr. North,
as to the general opinion of writers on
this subject. Chomel, who had great
t-xperience, and who had written, ex pro-
fesso, on the subject, pronounced haima-
temesis " une maladie grave." In the
first case related by Mr. North, he con-
sidered the mucous membrane of the
stomach and bowels in a state bearing
strong analogy to that which sometimes
obtains in purpura hemorrhagica. It was
agreed, on all hands, (with one excep-
tion,) that the blood issued from the
capillary vessels, by a sort of exhalation,
and that they were in a kind of atonic
condition at the time. The exception to
this opinion was enunciated by Dr. Ayre,
who believed that the blood, in all these
cases, came from the liver, and not from
the vessels of the stomach or bowels.
This pathology, however, wanted one
material point of support ? anatomical
evidence. Dr. Ayre had not traced the
sanguineous or melsenic lluid into the
ducts of the liver; but he had observed
that the premonitory symptoms of hsema-
temesis were the same as those which
preceded and accompanied other and un-
equivocal affections of the liver?while
the remedy which he had always found
effectual in removing the complaint was
calomel, the medicine which was also
most efficacious in hepatic disorders ge-
nerally. The worthy Doctor having some-
what inadvertently contemned the patho-
logy of Hippocrates and the ancients, on
account of their ignorance of anatomy,
and want of opportunities for post-mortem
researches, was taken to task by Dr.
Barry, who maintained that Dr. Ayre's
pathology was founded on precisely the
same principles as those of Hippocrates
?the symptoms and the effects of. reme-
dies. Dr. Ayre had observed a blanched
state of the liver and spleen, in some
cases where the intestinal haemorrhage
had proved fatal. This, however, was
evidently the consequence of the haemor-
rhage itself, and would have been the
same whether the blood came from the
liver or from any other part of the system.
But, whatever might be thought of
Dr. Ayre's pathology, (which certainly
appeared to us to be much too limited,)
the practice which he had found to be so
uniformly successful?namely, repeated,
small doses of calomel, till bile appeared
in the motions, and till the melcenic dis-
charges ceased, should not be despised.
Dr. Copland related some cases which
had been treated by the oil of turpentine,
both by the mouth and by enema, with
complete success. In these cases, there
was discharge of half-digested blood from
the bowels?but not the melrenic stools.
Dr. Johnson had seen three cases of idi-
opathic hainiatemesis, where no melsenic
discharges were present, and where there
was no evidence of organic disease, in
514 Periscope ; or, Circumspective Review. [Feb. 23
which half-drachm doses of sulphate of
.zinc suddenly arrested the haemorrhage
from the stomach, as soon as vomiting
took place. He would therefore be dis-
posed to adopt the same remedy, under
similar circumstances?but not other-
wise. The combination of melaena with
the haematemesis would induce him to
expect a morbid state, or, at all events,
a morbid action in the mucous membrane
of the intestines as well as stomach, and
consequently a condition not proper for
the emetic practice.
Dr. Barry drew the attention of the
Society to the superiority of local over
general bleeding, in cases of vicarious
haemorrhages. Thus, when hsemateme-
sis, from suppressed or interrupted men-
struation, occurs in this country, we
seldom think of attempting to restore the
natural or original discharge, by leeching
the labia, the hip-bath, &c.?or of ap-
plying leeches to the anus, where there
has been suppression of the hsemorrhoidal
discharge- Yet such is the practice all
over the Continent?and such practice
is rational. The doctrine of derivation
(it was. observed by Dr. Barry) was now
much in disgrace?but perhaps unmerit-
edly so, as there were few points of an-
cient pathology better founded in nature
or fact.
A gentleman, from the Middlesex Hos-
pital, related three cases of haematemesis
?two of which proved fatal?the third
was recovering. In all of these cases,
there was a melsenic discharge from the
bowels. This gentleman disagreed with
Dr. Johnson, in the opinion that the black
discharge from the bowels, in haemate-
mesis, was an indication of much dan-
ger. Dr. Johnson referred this gentle-
man to a numerical consideration of his
own cases, where two out of three died,
under this complication.
Upon the whole, it appears to us that
there was not sufficient distinction made,
by several of the members, between sim-
ple vicarious haematemesis and that dan-
gerous disease melasna. Although Dr.
Ayre did not admit that the disease for
which he prescribed calomel was true
melaena, (at least so we understood him,)
yet we apprehend that it is in this last
affection, rather than in simple haemate-
mesis, that the above-mentioned medicine
is efficacious. At all events, the cases
brought forward by Mr. North and others,
will render medical men more circum-
spect in their prognosis, than appears to
have been the case in the foregoing in-
stances.
2. Erysipelas.
The local treatment of erysipelas by in-
cisions, seems to come mox-e and more
into favour. Dr. Dobson, of Greenwich
Hospital, has transmitted a paper to Mr.
Lawrence (which was lately read at the
Medico-Chirurgical Society) in which is
detailed the method he has adopted for
ten or twelve years past, of making punc-
tures with a lancet, into the erysipelatous
parts, whether the erysipelas be cuta-
neous, phlegmonous, or oedematous.
These punctures vary in size and depth?
the object being to unload the vessels
both of blood and serum. The success
attending this practice appears quite as-
tonishing. He employs the remedy in
traumatic, as well as idiopathic erysi-
pelas?and also in that red, inflamed,
and hardened state of parts which sur-
rounds old ulcers in the extremities. By
these incisions, we learn that Dr. Dobson
has cured numerous cases of ulcers, that
had for years, been rebellious to all other
kinds of treatment. Dr. D. conjoined
active purgation of calomel and colocynth,
with subsequent diaphoretics.
Mr. Earle observed that, however bene-
ficial might be these local means in ery-
sipelas, as adjuvants to constitutional
treatment, yet still, in his experience,
the disease was, in a majority of in-
stances, connected with, or dependent on,
derangement of the general health. On
this account, he had found smart pur-
gation, especially with calomel and tartar-
emetic, conduce to recovery, in a very
remarkable manner. Local means were,
of course, to be used at the same time.
Dr. Johnson followed on the same side,
and contended that, in the majority of
cases, erysipelas on the surface was merely
a symptom of constitutional disorder. If
patients were attentively watched, there
would be found preceding phenomena
that indicated a derangement of the whole
system, as manifested by the tongue, the
sensations, the secretions, and the ex-
cretions. This being the case, it is pretty
certain that cases of erysipelas would, in
most instances, get well, by attention to
1828] Medical Jurisprudence. 515
diet and the bowels, with very common
local applications.
Mr. Lloyd, since the recent discussions
on erysipelas, had had several oppor-
tunities of testing the efficacy of the
Method recommended by Dr. Dobson?
small incisions with the lancet?and had
experienced the most beneficial results.
In one or two cases, where the local in-
flammation had resisted the enormous
venesection of /O or 80 ounces of blood,
at one bleeding, the erysipelas gave way
a series of punctures by the lancet.
This treatment had been employed by
him in erysipelas of the scalp?the ab-
domen?and the extremities. Mr. Lloyd
never, of course, neglected the consti-
tutional treatment, and always paid great
attention to the bowels. Mr. L. con-
firmed the statements of the author of the
paper (Dr. Dobson) as to the good effects
of local incisions in the neighbourhood
of old and obstinate ulcers. By this
auxiliary, he had recently cured an ulcer
of the lower extremities, which had re-
sisted all other modes of treatment. Mr.
Alcock observed, that this practice was as
o]d as the days of Ambrose Pare, and that
Lisfranc always employed it in La Pitiis.
Mr. Lawrence made some observations
on the local treatment of erysipelas, as
recommended by Dr. Dobson ; and then
stated the particulars of a case that had
recently occuiTed, of dissection-wound,
in the person of the present very intelli-
gent house-physician of the Fever-Hos-
pital?Dr. Dill. This gentleman, while
opening the abdomen of a female who
had died of puerperal peritonitis, re-
ceived a scratch on the back of his hand,
from the extremity of a sawn rib. The
consequences were, some uneasiness at
the time, and a pustule or vesicle the
next day, with tense and shining inflam-
mation of the back of the hand, accom-
panied by great pain. An incision, of
about an inch in length, from which a
great quantity of blood issued, gave great
relief?and this was promoted by the ap-
plication of leeches, and the immersion
of the hand in warm water. Dr. Dill
rapidly recovered. Drs. Tweedie and
Gregory corroborated this statement. It
is probable that, as far as the instrumental
treatment of erysipelas is concerned, the
mode of Dr. Dobson will supersede the
long and short incisions by the scalpel.
We have reason to know that the lancet
cuts heal generally by the first intention,
and consequently leave no scar, which
might not be the case with scalpel inci-
sions?and, about the eye-lids and face,
it is of some consequence to avoid sub-
sequent deformity. We learn that, in
some cases, where the eye-lids were
greatly swelled with erysipelatous inflam-
mation, the incisions by lancet gave
almost immediate relief?enabled the
patients to open their eyes,?and brought
the erysipelas quickly to a favourable
3. Medical Jurisprudence.
The following is the recent curriculum
promulgated by the College of Surgeons,
and to which allusion has been made in
the article on medical education in the
present number
"Regulations of the Council relating to the
Age and, Professional Education of
Candidates for the Diploma of the
College.
" I. The only schools of anatomy, and
physiology, recognized, are London, Dub-
lin, Edinburgh, Glasgow, and Aberdeen.
" II. Attendance upon the surgical
practice of an Hospital will be recog-
nized, pj-ovided such Hospital contain at
least one hundred patients.
" III. No person under twenty-two
years of age shall be admitted a member
of the college.
" IV. The following certificates will
be required of candidates for the diploma
of this college :
" 1. Of having been engaged six
years, at least, in the acquisition of
professional knowledge :
" 2. Of having regularly attended
three or more winter-courses of ana-
tomy and physiology; and two or
more winter-courses of dissections
and demonstrations; deliveredat sub-
sequent periods.
" Two courses of anatomy and
physiology in Edinburgh or Dublin,
which are of six month's duration,
and the accompanying courses of dis-
sections and demonstrations ivill be
considered as equivalent to the fore-
going attendance.
"3. Of having regularly attended
two or more courses of lectures on
516 Periscope ; or, Circumspective RevieyV. [Feb. 23
the principles and practice of sur-
gery ; one of which shall have been
delivered in a recognized school of
anatomy.
"4. Of having also attended the
following lectures, viz.
" Two courses on the theory
and practice of physic of three
months each, or one of six
months.
" One course on materia
medica, and botany.
" Two courses on chemistry,
of three months each, or one of
six months.
" Two courses on midwifery,
of three months each, or one
of six months.
" 5. And of having attended, du-
ring the term of at least one year,
the surgical practice of one or more
of the following Hospitals ; viz. St.
Bartholomew's, St. Thomas's, the
Westminster, Guy's, St. George's,
the London, and the Middlesex,
in London; the Richmond, Steven's
and the Meath, in Dublin ; and the
Royal Infirmaries, in Edinburgh,
Glasgow, and Aberdeen ; or during
four years the surgical practice of a
recognized provincial Hospital, and
six months, at least, the practice of
one of the above named Hospitals
in the schools of anatomy.
"V. Candidates under the following
circumstances, of the required age, and
who have been engaged five years in
the acquisition of professional knowledge,
will be admissible to examination, viz.
" Members, or licentiates in
surgery, of any of the legally con-
stituted colleges of surgeons in
the united kingdom.
" And graduates in medicine of
any of the universities in the
united kingdom, provided they
have attended lectures, the prac-
tice of an Hospital, and performed
dissections, as required in regu-
lation IV.
" VI. The required certificates shall
express the dates of the commencement
and of the termination of attendance on
each course of lectures, and dissections ;
and also of attendance on hospital-prac-
tice.
" VII. The required certificates shau
be delivered at the college ten days before
candidates can be admitted to examination.
" By order,
" Edmund Belfour, Secretary.
" 5tk day of January, 1828."
It is not here necessary to make any
comments on the new curriculum, since
the whole system is in need of re-construc-
tion.* But we may observe, that the
College is beginning to act on those
principles, which we have so long endea-
voured to inculcate?the union of medi-
cal with surgical knowledge. We have
shewn, on many occasions, the impossi-
bility and the absurdity, of separating
the study of surgery from that of physic,
and vice versa. The College of Surgeons
have now broken the ice, and driven their
crow-bar through the paper partition that
divided them from their brethren in Pall-
mall East! They have planted their
colours (and very properly too) in the
very camp of their rivals. They have
only to extend a little the medical flank
of their curriculum, in the next edition,
and enforce their standing orders by pro-
per musters and inspections (examina-
tions) and then the surgeons of this
country, will be, bona fide, physicians?
or rather they will be, what Hippocrates
himself was?general practitioners !
The College of Physicians has only to
persevere for a few years more in riding on
stilts, and the whole practice of the pro-
fession will be in the hands of surgeons.
Let them carefully avoid all attempts to
follow the dictates of reason and common
sense?let them continue to prescribe
Greek, Latin, and Mathematics, instead
of anatomy, surgery, and pathology, and
the public, as well as the profession,
will soon see the absurdity of consulting
those, whose education fits them better
for a cloister or a monastery, than for
clinical practice. It would be useless for
us to offer their high mightinesses any
counsel on this occasion. Cardinal Wol-
sey is their pillar of light?and his priest-
craft is their favourite physic.
* We shall comment on some items of
this curriculum in our next fasciculus.
1828] Gangrenous Erysipelas, &c. 517
HOSPITAL PRACTICE.
1. ROYAL INFIRMARY OF EDIN-
BURGH.
Gangrenous Erysipelas. Amputation
?Death.*
We were somewhat startled, on looking
at the table of contents of the Lancet,
to find the following heading. " Case
?f gangrenous erysipelas, in which am-
putation was performed with success."
On turning to the case in question, how-
ever, all our pleasing anticipations va-
nished into " air?thin air," for we found
that, though the amputation might have
been performed with success, the patient
had most certainly died.
Case. D. H. aged seventy-two, ad-
dicted to drink, was admitted, Jan. 4Oth,
under the care of Dr. Hunter, with gan-
grenous erysipelas of the arm. The pa-
tient appeared to be moribund?the whole
limb, up to near the axilla, was enormously
swelled, of a dusky red colour, and quite
livid over the wrist and metacarpal bones,
whilst here and there were several large
vesications. He stated that, ten days
previously, he had received a bite on the
forefinger from a boy, and that nothing
had been applied but poultices.
Five or six incisions, about two inches
in length each, were made by Dr. Hunter
in the arm, and he was ordered wine in
small doses, until the pulse rose, with a
fermenting poultice to the limb. On the
next day, the pulse was stronger, but ir-
regular?tongue coated?bowels confined.
JVine to be continued. Quin. Sulph. gr. ij.
Mist, camph. ?j. 4tis horis. 12th. Pulse
rather fuller?bowels not yet opened?
wounds gaping, and filled with a beer-
coloured fluid. 01. ricini, 3i'j- s tat mi.
Camphor emulsion to be omitted?wine?
quinine?-fermenting poultice.
We shall not pursue the diurnal details
of this case, which " drag their slow length
along," with the most tiresome verbosity.
On the 13th, the inflammation had exten-
ded on the posterior aspect of the arm,
but, on the 14th, it was diminishing, and
three of the wounds had begun to suppu-
rate. On the 15th, he was no better,
but the report states that a line of sepa-
ration was beginning to fonn above the
elbow, though it was not a very decided
one. On the 16th, it was determined, in
consultation, to amputate the arm, which
was done, " a little above the middle of
the humerus," by Dr. Hunter. The mus-
cles of the fore-arm were flaccid, but not
disorganized, and the cellular membrane
over the carpus and metacarpus was des-
troyed, some matter being thrown out
amongst the sheaths of the extensor ten-
dons. No mention whatever is made of
the state of the stump, or whether the
operation was performed in sound or un-
sound parts ! Next day the patient was
more comfortable, but somewhat incohe-
rent ; tongue moist and cleaner. Qui-
nine to be discontinued, and three ounces
of white wine in whey, to be given with
barley water and beef tea.
We shall not bore our readers with the
prosy details which Scotus has given de
die in diem; suffice it to say, that no at-
tempt at union took place upon the stump,
and that the patient never rallied from
the operation. The pulse was never be-
low one hundred?the tongue coated with
yellow mucus?the mind more or less
affected. On the 20th, hq became af-
fected with diarrhoea, which, however,
was checked by infusion of roses, with
sulphuric acid, and chalk mixture. On
the 21st, the pulse was feeble and inter-
mitting, he became affected progressively
with rigors, hiccup, &c. and, on the 24th,
he died. In the account of the dissection,
it is merely stated that the heart was
" slightly diseased," and the left lung
covered with an old false membrane.
Remarks. Of this case, reported as it
is, we can make, to use a vulgar phrase,
?'neither top nor tail." First of all we are
told that the whole limb was enormously
swelled, and of a dusky red colour " from
near the axilla to the ends of the fingers."
On the 13th, the inflammation had " ex-
tended on the posterior aspect of the arm,"
and yet, on the 15th, a line of separation
Avas forming, just above the elbow!!
Now in our younger days, some twenty
years ago, or more, we well remember
being taught, that the line of separation
was a boundary, formed by a sloughing
* Lancet, No. 232.
518 Periscope; or, Circumspective Review. [Feb. 23
process, between sound and unsound parts,
between the living matter and the dead.
How, then, we would ask Scotus, or any-
body else, could a line of separation form
above the elbow, when his own report
affirms, that the disease had extended to
near the axilla. It would be an odd kind
of separation, indeed, which divided the
dead matter from the dead !
Secondly, we would ask Dr. Hunter,
upon what principle, or for what reason,
he made six incisions into the limb upon
the patient's admission ? Did he expect
to find matter, or did he expect to pre-
vent its formation ? If he did either, we
think he did wrong. This was not a case of
phlegmonous erysipelas, or of erysipelas
phlegmonodes. The tint, we are told,
was not bright, but dusky red ; the skin
was not tense and glistening, but covered
with gangrenous phlyctense; the pulse
did not indicate action, but was " frequent,
feeble, and irregular." Was this a case
for incisions ? ? We should say it was a
case for bark and opium, and that with
no sparing hand.
Thirdly, we cannot conceive why the
operation was performed about the middle
of the arm. Surely the object of the sur-
geon must have been to get fairly above
the disease, and how this was to be ac-
complished by amputating in the arm,
whilst the erysipelas had extended almost
to the axilla, we declare we cannot, for
the life of us, comprehend.
Fourthly and lastly, we doubt the pro-
priety of performing the operation at all;
for, as far as we can judge, a man of se-
venty-two, who had drunk freely, who
was labouring under the most terrible
form of erysipelas, and who was in the
very worst condition imaginable, either
to originate or maintain the reparative
process, was no subject for the amputat-
ing knife, however, or wherever, applied.
We know not, for we have no means of
knowing, whether this report of Scotus
be correct or otherwise; but we will say,
that whilst the exact quantities of wine
taken, and stools voided, are doled out by
the yard, the most important particulars,
as the state of the parts in which the
operation was performed, the appearance
of the face of the stump, &c. are passed
over in silence. The consequence of all
this is, that the reader gets up from the
perusal of the case, just as wise as when
lie sat down to it;
2. MIDDLESEX HOSPITAL.
Mu. Belt, on the Question of
Amputation.*
The question of primary and secondary
amputation, in cases of gun-shot wound
or compound fracture, one would imagine
to be pretty well set at rest. Mr. Bell,
however, has dedicated two clinical lec-
tures to the subject, and, what is more,
contrived to make them exceedingly in-
teresting. We notice these observations
more for the purpose of commenting on
one or two points, than with any inten-
tion of detailing them at length.
Case. A soldier, aet. 27, whilst drunk,
fell in the street, and the wheel of a loaded
waggon passed over his leg. He was ad-
mitted soon afterwards into the Middle-
sex Hospital with compound fracture of
the leg, the tibia protruding, and simple
fracture of the femur in two places, viz.
at its centre, and near the trochanter
minor. The operation was deferred till
next morning, to allow of his recovering
from the intoxication, when it was per-
formed high in the thigh. Mr. Bell, be-
ing one of those gentlemen who believe
it impossible effectually to compress the
artery at the groin, every precaution was
taken against hasmorrhage. First, there
was the tourniquet applied; secondly,
there was a gentleman with his thumb
on the femoral artery; and, thirdly, to
keep out the blood from below, there was
" a fillet tied firmly round the thigh, be-
low where it was intended to make the
incision." No doubt these were proper
precautions, and must have been excel-
lent lessons for the pupils of the Middle-
sex Hospital; but, probably, if the ampu-
tation had been performed in the cock-
pit, or, as Mr. Bell once expressed it,
" on a pack-saddle at the top of the
Pyrennees," the surgeon would have con-
trived to do without tivo of them, at any
rate. An incision was then made on the
fore part of the thigh, cutting across the
main artery, which bled freely. It was
accordingly taken up by the tenaculum
and tied, as was the profunda. The ope-
ration was concluded by making two
semicircular flaps, one on the inner, the
other on the outer side of the thigh, and
removing the bone. The patient hitherto
* Med. Gazette, Nos. 8?10.
1828]
Mr. Bell on the Question of Amputation. 519
has gone on pretty well since the ope-
ration.
Upon this case, Mr. Bell makes several
remarks. He observes that there are
two kinds of compound fracture. A man,
forinstance,leaps from his gig, andhreaks
his leg. The limb is twisted, the bone
protruding, the case apparently a bad
one; " but the surgeon extends the limb,
reduces the bone, lays it carefully out upon
& pillow, prevents the rising inflamma-
tion by cold lotions, or iced water,?and
what is the amount of the injury?" The
great vessels and diiferent textures are
little injured, and the patient will pro-
bably do well. If, however, the limb has
heen crushed, the muscles rent?blood
extravasated?the great vessels torn, the
appearances to the eye may be the same,
hut the issue of the case will be very dif-
ferent indeed. The above was an instance
of the latter kind of injury, and had not
amputation been performed, gangrene,
or other fatal termination, would, in all
probability, have been the consequence.
Passing over Mr. B's reasons for ampu-
tating high in the thigh, we come to the
question of haemorrhage. Mr. John Bell,
it is well known, denied that the femoral
artery could be effectually compressed.
It is a diiferent thing, says he, to stop
the pulsation, and check the flow of
blood. Now, in spite of the authority of
Mr. John Bell, surgeons have an idea
that they can do both, without any very
great difficulty. Sir Astley Cooper, as
the story goes, demonstrated this to a
young gentleman in the operating theatre
of Guy's Hospital, by lifting his thumb
from the vessel, and directing the stream
of blood, we will not say upon him, at plea-
sure. Mr. C. Bell argues that this prac-
tical joke of the worthy Baronet's proves
nothing at all; tfie blood being stopped
not by the thumb upon the artery, but by
the hands grasping the thigh, and so
compressing the collateral vessels. This
is ingenious reasoning, but we have seen
the circulation completely arrested, with-
out any grasping of the thigh at all, the
pressure being made upon the artery by
the key-compress.
We contend, indeed, that in the high
amputation of the thigh, the tourniquet
is at times a positive disadvantage, for it
prevents the due retraction of the muscles
and integuments, is in the surgeon's way,
and instead of arresting haemorrhage,
rather tends to favour its occurrence.
This may appear paradoxical, but when we
consider that the artery, in this situation,
does not lie closely on the bone, but in a
kind of hollow between the sartorius and
pectineus, we see at once, that by the
contraction of these, and the other mus-
cles, the tourniquet is ineffectual in pre-
venting the circulation through the artery,
and through the profunda, whilst it is
quite, or nearly quite, effectual in pre-
venting its return through the veins.
The consequence is obvious?loss of blood.
These remarks apply of course, only to the
high operation, for the surgeon who makes
a boast of never requiring or desiring the
tourniquet in any case, is only depriving
himself, most unnecessarily, of a very
valuable assistant.
With regard to the time of operating
in these severe cases of gun-shot wound
or severe fracture, Mr. Bell very judi-
ciously observes that, after the patient
has once recovered from the shock of the
accident, and has revived from the state
of depression, " so that he sees by the
shattered condition of his limb, that it
must be lost, then the operation ought to
be performed, and the sooner the better."
Mr. B. pays a merited compliment to the
surgeons of the British Navy, but singu-
larly enough, omits their brethren of the
Army, which is certainly not at all fair to
the latter.
The next case which occupies the at-
tention of the lecturer, is one of distorted
limb, where the patient wished to have the
defective member removed. This wish
of a patient's, Mr. Bell thinks, c?eteris
paribus, should go for nothing in deter-
mining the question ; as cases have re-
peatedly occurred, when in compliance
with such requests, the limb has been
amputated, and the person died. A tai-
lor, for instance, had so crooked a leg,
that he could not sit in the proper posi-
tion on the board. He suffered ampu-
tation and died. The case under con-
sideration, was one in which the motions
of the knee-joint were much impaired in
consequence of an old and ill-set fracture ;
conjoined with which there were ulcers
upon the leg. Mr. Bell taking into con-
sideration the fixed determination of the
man to have his leg off, either in that
hospital or elsewhere, the utter lameness,
and the ulcers which were continually,
and would be continually breaking out,
520 Periscope; or, Circumspective Review. [Fbb. 23
at last consented to amputate. Symp-
toms of tetanus came on, but disappeared ;
a bad state of the stump supervened, and
at the end of five weeks the patient sank.
Mr. Bell makes one observation, in
which we do not quite agree with him,
namely ft that he looked upon the ulcers
of the leg as favourable to amputation."
Now it certainly seems to us, that these
old sores indicate a state of system far
from favourable to any great operation,
and in one or two cases where amputation
was performed for such sores, we have
witnessed an unfortunate result.
Case 3. A man ajt. 54, having received
a slight scratch upon his little finger, ery-
sipelas shewed itself, and was relieved by
incisions into the arm and hand, giving
vent to serum and pus. The erysipelatous
inflammation, however, l'ecurred at inter-
vals, an abscess exposed the joint of
the thumb, and another surrounds the
elbow-joint. In this case, Mr. Bell is
adverse to the amputation, because the
slight nature of the original injury, and
the occurrence and recurrence of the
erysipelatous inflammation are evidence
of something defective in the constitution,
which might, and probably would, equally
shew itself in the stump. A young woman
in the Middlesex Hospital, was a remark-
able instance of this liability to erysipelas.
If leeches were applied, an erysipelatous
blush extended up the thigh, the same
with blisters, caustics, &c. so that the
girl nearly died. At last amputation was
performed (for disease of the knee-joint)
a bad state of the stump was the con-
sequence, ushered in by erysipelatous in-
flammation, and she narrowly escaped
with life.
We have been led farther into these
lectures of Mr. Bell's than we intended
to have gone, but really the interest of
the matter, and the engaging manner iu
which it is treated, cannot fail, we think,
to be a sufficient apology.
3. STEEVENS' HOSPITAL, DUBLIN.
Vapour-Bath in Tetanus.
Dr. Marsh has published some brief no-
tices of the effects of vapour-bath in this
dreadful, and generally fatal malady. The
first case was that of a boy, five or
six years of age, who was brought into
Steevens' Hospital, Dublin, labouring un-
der tetanus. The paroxysms were severe
and frequent, and the disease had come
on after an injury in the great toe. Ca-
lomel in large doses, with other purgative
medicines, having failed to act on the
bowels?and opium not giving any relief,
the vapour-bath was suggested, and the
boy was subjected to it, at a heat not
above 90 degrees. He remained in the
bath six hours. The paroxysm became
less violent and less frequent. A third
of a drop of croton oil was given every
third hour, which operated violently after
the fourth dose. Belladonna, and ol.
succini, were rubbed along the spine.
The bath was daily employed, for several
hours each time, and the boy slowly re-
covered. It was remarked that, on re-
moving the little patient from the vapour-
bath, the sore, which had previously been
foul and unhealthy, began to improve in
appearance, and ultimately assumed a
clean granulating aspect.
The next case was also one of traumatic
tetanus, but unsuccessful. " The uniform
effect of the vapour-bath was to abate the
violence of the paroxysms, without, how-
ever, influencing, in the slightest degree,
the perhianent rigidity of the muscles."
In the third case, the patient was an
adult, who was under the care of Mr. Cu-
sacb, in the aforesaid hospital. Calomel
and opium had been given till ptyalism
was established ; but apparently, without
any good effect. The vapour-bath was
then tried, and steadily persevered in,
till every symptom of the disease gra-
dually gave way. In this and the other
cases, the patients were enveloped in a
flannel bag, to which, at the lower part,
a small tin boiler was attached. Under-
neath this, a spirit of wine lamp main-
tained an abundant supply of vapour, the
temperature being regulated by a thermo-
meter,introduced into the flannel envelope.
Thus, then, two patients were saved
out of three, and one of those saved la-
boured under traumatic tetanus. We
conceive that the long-protracted vapour-
bath, at a low temperature, as above des-
cribed, is infinitely preferable to liquid
baths in the common way, or vapour-
baths at a high range, where, of course,
the stay in the bath must be comparatively
short. This plan does not interrupt or
interfere with the exhibition of medicine,
and we strongly recommend its adoption.
1828]
Injury of tiie Head. 521
4" ST. BARTHOLOMEW'S HOSPITAL.
Injury of the Head?Appi.ication of
the Trephine.
any one set of cases is more puzzling
'atl another in surgery, it is??" Injuries
the Head." We verily believe that,
a student were to go the round of the
Afferent surgical lecturers in this metro-
P?lis, he would scarcely find any two
freeing in their practice in these cases.
?lr Astley Cooper tells us, that it makes
the difference in the world, whether
the cranial fracture be simple or com-
pound. Mr. Travers and Mr. Brodie seem
J? say that it makes no difference at all!
Here, then, upon a mere matter of expe-
rience, for the comparative danger of one
case over another can be known only by
experience, sound practical men are at
*?ggerheads. In medicine, alas ! there is
Nothing certain, but death and the doc-
k's bill.
Case. W. B. act. 30, was thrown from
a cart, picked up insensible, and taken
|o a surgeon, who bled him, and dressed
"is head. He regained his senses, and
yomited frequently, but soon fell again
1,1 a state of stupor, and next morning,
?Jan. 23d, was admitted under the care of
Mr. Earle. At this time, he was drowsy
T""":incoherent?pupils dilated, but ansvver-
lrig to the light?pulse small and irregular
'?extremities cold. Over the upper and
lateral part of the left parietal bone, there
^v'as a lacerated scalp-wound, but the bone
did not appear to be denuded. He was
ordered calomel and jalap, and, at 4, p.m.
there being some re-action, he was bled.
the evening, 20 ounces of blood were
taken from the temporal artery, with
some little relief, and, at 12, p.m. was
tied again to ?xvj. ""Next day he had
difficulty of articulation, pulse full and
Sequent, pupils much dilated. V.S. ad
Sxvj. On the 25th, there was paralysis
?f the right side of the face, and he passed
the urine and faeces involuntarily. 26th.
Partial paralysis of the right side of the
body?coma. The wound was sloughy,
and the bone beneath bare for about an
iflch, and, apparently, dead. In consul-
tation, it was determined to apply the
trephine. Upon removing the bone, nei-
ther blood nor matter was found upon
the dura mater, but this membrane had
a blueish tint, as if there were blood de-
posited beneath, and no pulsation of the
brain was to be felt. He appeared to re-
cover a slight degree of sensibility after
the operation, but soon relapsed. The
pupils were greatly dilated?the breathing
stertorous?he became violently convul-
sed, and, at 2, a.m. of the 28th, expired.
Dissection. Dura mater not inflamed,
but on removing this membrane, a quan-
tity of blood was found effused over the
whole cerebrum. The left hemisphere,
opposite the point where the bone "had
been removed, was flattened, by the de-
position of a quantity of dark clotted
blood, and the posterior surface of its
middle lobe was coated with grumous
blood. On making a section of this lobe,
it was found to have undergone the pro-
cess of ramollissement to the extent of
about two inches. Besides this, a frac-
ture extended from the left temporal
bone, through the anterior inferior angle
of the parietal, passing across the groove
for the meningeal artery. Between the
dura mater and bone, at this part, no less
than three or four ounces of blood had
been extravasated.?Gazette, No. 10.
Mr. Earle, according to the report,
considered that, in the earlier part of
the case, the symptoms of concussion
were present, while, latterly, those of
compression supervened. On this point,
we cannot exactly agree with Mr. E. for,
as far as we have seen of concussion, the
patient, though drowsy, is not generally
incoherent, nor is the pulse "small and
irregular." Besides, after having been
bled by the surgeon, before his admission
into hospital, the patient recovered his
senses, but relapsed again immediately.
This certainly is not the case with con-
cussion, for, having once recovered from
this state, the patient can scarcely be
said to fall into it again, but rather to
suffer from the inflammatory action, set
up as a consequence or sequela of the
concussion. It appears to us, that this
man laboured under tolerably well-marked
symptoms of extravasation throughout,
although latterly they were aggravated,
partly from the re-action in the system,
and partly from the disoxganizing process,
which terminated in the " ramollisse-
ment" of the brain. The practice pursued
by Mr. Earle appears to have been highly
judicious.*
* This article had been sent to press,
Vol. VIII. No. 16. 3 L
522
Periscope; or, Circumspective Review. [Feb. 23
We shall here introduce a case which
occurred at St. George's Hospital, and is
illustrative of the difficulty which exists
in ascertaining the exact nature of the
injury which the brain has received.
A boy was brought into the hospital,
Aug. 22d, at half past one, p.m. in a state
of total insensibility, having fallen from
a height of 30 feet. There were two ex-
tensive scalp-wounds upon the right side,
but the pericranium was entire, and no
fracture of the bone could be discovered.
The pupils were rather contracted and
sluggish?pulse almost natural?surface
cool?breathing much like that of a per-
son asleep, and without stertor. When
his name was halloo'd loudly in his ear,
he was perfectly unconscious of it, but,
whilst some bleeding vessels were being
when we received No. 233 of the Lancet,
containing a most abusive tirade against
Mr. Earle, for the practice which he pur-
sued. There is so large an admixture
of slang, " cock-sparx-ows"?" bats"?
" vampires," &c. &c. that, for some time,
we could not persuade ourselves but that
we were perusing the account of a mill,
at Moulsey Hurst, between " Barney Aa-
ron" and the " Chelsea Snob," or some
such celebrated personages. The sapient
critic in the Lancet sports we know not
how many notes of admiration, at Mr.
Earle's considering the symptoms enu-
merated above as those of compression.
Now we have said, and we do maintain
it, that the symptoms ivere those of com-
pression, almost from the very commence-
ment of the case, exasperated certainly,
at last, by the inflammation going on in
the substance of the brain. If the symp-
toms did not indicate compression, what,
in Heaven's name, did they indicate ?
Concussion? The man in the mask must
be even a greater goose than we take him
for, if he asserts that difficulty of articu-
lation?paralysis of one side?and invo-
luntary discharge of faeces and urine, are
evidences only of concussion. Whether
the trephine should have been applied
earlier than it actually was, may admit of
doubt ?, but the man who can rate a sur-
geon, as Mr. Earle is rated, for applying
that instrument in a case, where dissec-
tion shewed " three or four ounces" of
blood over the dura mater, and a consi-
derable quantity wider it, ought to be
ashamed of himself.
secured upon the scalp, he struggled vio-
lently, and moaned.
In the evening, he grew restless and
fidgetty, and slight re-action came on-
He was still insensible, and the pupil3
were dilated, particularly that of the righ1
eye, but without any strabismus, or con-
vulsive affection, in any of the muscles.
V.S. ad gviij.
23d. Evidently sinking. The surface
is cold?the pulse scarcely to be felt?*
pupils act on the approach of a candle*
but the right is still dilated. Towards
evening he became rather convulsed, and;
at 6, p.m. quietly expired.
Sectio Cadaveris. Mr. Brodie, under
whose care the patient was, imagined
that the symptoms depended upon extra-
vasation beneath the dura mater. On
raising the calvarium, no blood whatever
was found, either above or below this
membrane, and the hemispheres were
sliced down to the level of the corpus cal-
losum, without any extravasation making
its appearance. In the substance of the
cerebrum, a little to the left of the septum
lucidum, was a small spot, softened, and
containing a little blood. This was also
the case with the left optic thalamus, and
in the lower part of the posterior lobe of
the cerebrum, was a cavity, about the size
of an almond,enclosing extravasatedblood.
A fissure was found running across the
optic fossa of the sphenoidal bone, and
the liver was of that granular texture found
in dram-drinkers.
It would be difficult to say how far the
symptoms, in this case, were owing to
compression, and how far to concussion.
The want of stertor?the coldness of the
surface, and the almost natural state of
the pulse, would seem to evidence the
latter, whilst the unequal dilatation of
the pupils, and the general intensity of
the symptoms, might rather be referred
to the former. These are the mixed cases
which occur in practice, and bother the
student, who has got by rote all the pretty
little distinctions, so clearly pointed out
in his elementary books.
5. ST. GEORGE'S HOSPITAL.
Peculiar Affections of the Cra-
nial Bones.
At page 489 of our last Fasciculus wc
1828] Peculiar Affection of the Jaw. 523
?}ave noticed some observations of Dr.
^bercrombie's and Mr.O'Halloran's upon
this head. Two cases have lately oc-
curred at St. George's Hospital, nnder
the care of Mr. Rose, which might well
have been appended to the article in
Question.
Case l.? A woman was admitted for
bronchocele, but there was casually ob-
served a tumour on the head. It had
been punctured three weeks previously,
a,id into the puncture a probe was intro-
duced, and veiit given to some glairy
serum. Next morning rigors and severe
constitutional disturbance came on, an
erysipelatous blush appeared upon the
leek, and on the 3d day she died. On
dissection the tumour was found to be of
Malignant structure, and to pass through
a circular opening in the parietal bone,
and take attachment (though not a firm
?ne) to the dura mater beneath. This
Membrane was quite sound, and there was
no reason to believe that the tumour had
originated from it.
Case 2. This patient we had an op-
portunity of seeing ourselves, and the
Particulars, we believe, have not been
Published elsewhere.
Caroline Hollyoake, set. 20, was ad-
mitted Nov. 7th, with extensive disease
of the right parietal bone. The outer
table was completely exposed for a space
about the diameter of a crown-piece, but
longer, and the edges of the scalp around
Were curiously tucked in. The bone was
rough and blackened, and around it a
line of separation had been commenced,
but was ineffectual in checking the dis-
ease, for the probe passed beneath the
scalp, and discovered dead bone for some
distance around. The coronal suture
crossed the exposed bone in front, and
between its seiTated edges, which were
iwore apart than they should be, pus was
seen undulating in correspondence with
the pulsations of the brain. The menses
had always been irregular, aud for the last
three years had ceased entirely. About
a year previously she began to be affected
with pain in the head, and hysteria, and
she first noticed matter amongst the hair,
nine months prior to admission. An ab-
scess appears to have formed, and the
part of the scalp to have been destroyed
by ulceration, Besides this disease of
the cranium, there was likewise dead
bone in the lower jaw. There was no rea-
son whatever to suspect a syphilitic taint
?she had never received any blow upon
the part?nor could she account for the
affection in any way. On the 13th, Mr.
Rose removed a portion of the blackened
outer table by an elevator, and on the
29th he divided the scalp, and removed
a still larger portion by means of Hey's
saw. Large flabby granulations were ex-
posed beneath, which pulsated strongly j
but whether they arose merely from the
diploe, from the dura mater, or partly
from one and partly from the other, it
would be difficult to say. The patient
was going on pretty well?the pulsation.
had distinctly lessened, and her health
was certainly not suffering much, when
she was persuaded by her stupid friends
to leave the hospital, lest she should be
made to undergo any more operations.
At this time the probe passed for some
distance across the os frontis, and there
was altogether a very large amount of
diseased bone on the right side of the
head. What was the cause of all this it
would be difficult to say, though it must
be owned that the girl was of a puny,
scrophulous habit of body. It is curious
how little disturbance these affections
cause to the sensorium. In the first case,
the patient was scarcely aware of the ex-
istence of the tumour, and, in the second,
there was no paralysis, no affection of the
mind whatever. Mr. O'Halloran objects
to the use of the trephine, but upon what
grounds we know not. If there is a mass
of diseased bone in the skull, particularly
if the outer table only is affected, it
surely must be an object to get rid, as
quickly as may be, of this dead and irri-
tating body. If, indeed, there be symp-
toms of irritation to the brain, as convul-.
sions, paralysis, epilepsy, &c, then we
conceive that the surgeon, cseteris pari-
bus, has no choice in the matter, but is
imperatively called upon to remove the
affected bone.
* Loud. Med. Gazette, No. 1.
524 Periscope; ok, Circumspective Review. [Feb.23
6. ST. THOMAS'S HOSPITAL.
On Non-union of Fractured Bone.
By Mr. Amesdury.
A very interesting paper upon this sub-
ject was read before the Medical Society,
at Bolt-court, on Monday night, by Mr,
Amesbury. He detailed at length,acase,
Which had occurred under the care of
Mr. Green at St. Thomas's Hospital, in
which several methods of treatment had
been unsuccessfully employed, and the
patient ultimately had the limb ampu-
tated.
A strong, healthy sailor, cetv. 36, was
admitted March 11th, 1827, with fracture
of the thigh about its middle-third, which
had existed for twenty-four weeks, and
been treated in a very ineffectual manner
at a Portuguese hospital. The fracture was
oblique, very loose, and the lower portion
of the bone was drawn up along the inner
side of the upper one, to the extent of
two inches and a half, but extension re-
stored the limb to its natural length.
By the politeness of Mr. Green, Mr.
Amesbury was allowed to apply his appa-
ratus, so as " to retain the limb of its
proper length, and press the fractured
surfaces strongly together." Much pain
followed the application of the apparatus,
and continued more or less for ten weeks,
when it was removed, but no union had
taken place. A seton was next passed
between the ends of the broken bone,
extension being at the same time kept up
by fixing the foot and the pelvis, and
applying a splint along the outer side of
the limb. On the 19th day after its in-
troduction, it was necessary to remove
the silk, in consequence of matter having
burrowed beneath the fascia, and af-
fected the constitution somewhat severely.
Thickening of the soft parts had taken
place, but nothing more. During the
suppuration which followed, pressure and
extension by means of splints were assi-
duously kept up, but without effect, and
at the expiration of nine weeks from
the withdrawal of the seton, Mr. Green
proceeded to cut down upon the ends of
the bone. A semi-circular incision was
made in the front and outer side of the
thigh, through part of the rectus and
vastus externus, and the bone exposed,
the ends of which were found to be
connected by a dense capsule. This
wn* removed from the upper portion of
the bone, but in consequence of the under
portion being drawn up on the inner
side of the former, it was found impos-
sible to get at the capsule connected
with it. Half an inch of the upper
portion of the bone was sawn off, and
discovered to be soft and spongy from
interstitial absorption.
After a time the splints were again
very carefully applied, but at the ex-
piration of nine weeks, no union ha%Ting
taken place, the limb was amputated at
the poor fellow's own request. On ex-
amination of the bone, the capsule was
found to be again complete, the greater
part of it being as thick as the capsule
of the hip-joint, and its inner surface
smooth like a synovial membrane. The
ends of the bone were rounded, and
where they came in contact, flattened
and covered with a libro-cartilaginous
membrane resembling the intervertebral
substance.
Upon this case Mr. Amesbury made
some very apposite remarks. In41 cases
out of 45 which he had witnessed, the
non-union was attributable, in his opi-
nion, as in the one detailed, not to weak-
ness of constitution, but to imperfect
adaptation of mechanical means. In
eighteen cases, Mr. A's plan has been
successful, most of them having existed
ununited above six months, one nine, one
ten, one eleven, and two fourteen months
before coming under his care. In two
cases only, this being one,* has Mr.
Amesbury failed. In all very loose frac-
tures of long standing, Mr. A. would be
disposed, after an unsuccessful trial of
pressure and rest by proper apparatus,
to cut down upon the ends of the bone,
remove by the knife the new capsule, and
stimulate the ends of the bone either by
a wash, or by the application of a caustic.
This, with the subsequent assistance of
due mechanical support, Mr. A. consi-
dered as affording the best chance of cure
in these very troublesome cases.
* The other, if we are rightly informed,
occurred at St. George's Hospital. We
shall take an early opportunity of giving
the particulars.
*828] Mr. Lawrence's Letter.
525
No. 1. Mr. Lawrence to Dr. Johnson.
18, Whitehall-place,
13th Feb. 1828.
Sir,?A friend of mine, who reads your
periodical publication, has sent me the
two last Fasciculi, calling my attention
to two statements which they contain
respecting myself. As you address to
me, in one of these, a kind of challenge
either to admit or deny it, and as both
are contrary to fact, I think it necessary
to notice them ; and I beg that you will
insert this letter in your next Number,
that my contradiction may reach those
who have seen your erroneous assertions.
In your comments on the case of Mrs.
Denmark, you say " we have been in-
formed, on the very best authority, that
on the very day after the operation, and
afterwards on the 10th or 12th day, con-
sequently before the ligature came away,
the pulse was distinctly felt by Mr.
Lawrence and others in the right arm.
If this information be incorrect, Mr.
Lawrence can easily contradict the state-
ment, when we will give our authority, who
also felt the pulse."?Fascic. II. p. 469.
I did not see Mrs. Denmark on the day
after the operation, nor within a week of
that time. I have seen her only once
since she was operated on, namely, about
the 10th day. I then visited her with
Dr. Tweedie, at the invitation and in pre-
sence of Mr. Wardrop, of whom she was
a private patient. I do not remember
the details of her symptoms at that time,
having no inducement to make minutes
of a case under the care of another gen-
tleman : but the impression on my mind
is, that a feeble pulsation could be felt in
the right radial artery. I am not certain
whether I ever saw Mrs. D. before the
operation; if I did, it was without having
the opportunity of inquiring minutely
into her case, or forming an opinion res-
pecting the treatment that it required.
In describing the case of J. Nowlan,
you say " on the 13th day he was better,
but the eye-ball was found to be greatly
protruding, which was attributed, by
Messrs. Lawrence and Wardrop, to a
powerful compression on the brain, exer-
cised by an aneurismatic state of the
vessels of the dura mater, communicating 1
through the skull with the tumour on the 1
head.?Fascic. III. p. 500. <
I never entertained nor expressed any
snch opinion.
Having thus talcen the trouble of con-
tradicting two mis-statements, because
they involve others as well as myself, I
beg that your readers will not consider
that I admit the correctness of those re-
presentations, which I leave uncontra-
dicted. Your report of the proceedings
at the Medical and Chirurgical Society,
contained in the same Fasciculus, which
ascribes to me the absurdity respecting
the case of J. Nowlan, would alone render
it necessary for me to enter this protest.
I shall make no remark either on the cor-
rectness of that report, or on the kind of
taste and feeling which it displays. The
former will be best estimated by those
who were present at the meeting, while
I doubt not that the latter will be properly
appreciated by your readers generally.
I remain, Sir,
Your obedient servant,
William Lawrence.
Dr. James Johnson.
No. 2. REPLY.
Let us now see what foundation there
is for all this supercilious and taunting
exposure of supposed mis-statements.
First. In respect to Mrs. Denmark's
case, the pulse teas felt in the right arm
on the very day after the operation, by
Dr. Barry and others, (who have made no
secret of it)?on the 10th clay, by Mr.
Lawrence himself, according to his own
admission. The facts, therefore, res-
pecting the pulse, are strictly correct?
though by not exactly assigning to the
observers the precise dates of their res-
pective observations, we have made Mr.
Lawrence (in the place of " others") ap-
pear on the second, instead of the tenth
day after the operation. We say again,
the facts respecting the pulse arc strictly
correct; and the whole of this mighty
error consists in putting " Mr. Lawrence"
before " others," in the statement. We
give Mr. L. all the glory he can claim,
tor the detection of such " erroneous
ASSERTIONS."
Secondly. Mr. Lawrence evidently
thinks he has branded its, beyond redemp-
tion, with the stigma of mendacity, in
the case of J. Nowlan. Let us see what
the " invaluable journal," the Koran
of the party, says upon this occasion.
526 Periscope; or, Circumspective Review. [Feb. 23
In the Report from the Hospital of Sur-
gery, (Lancet, No. 214, p. 24-5,) we find
the following passages :?
" On the thirteenth day, the stupor and
delirium had subsided, but the blindness
was undiminished, and the eyeball v;as
found to be protruding from the orbit, with
(Edematous effusion under the conjunc-
tiva, and in the palpebrse.
" These singular and distressing symp-
toms (including, of course, the protrusion
of the eye? ball?Ed. of Med. Chir. Review)
gave rise to much speculation, as to the
cause of their occurrence. Some physi-
ologists ascribing them to a want of sen-
sibility in the brain and its nerves, owing
to a deficiency in the supply of its stimu-
lus the blood, and resembling that state
produced in the lower extremities of the
inferior animals, by a ligature of the
aorta; or that loss of sensibility in the
fingers of man, arising from an obstructed
subclavian or humeral artery. Mr. Ward-
top and Mr. Lawrence ivere inclined,
hoivever, to take a very different view of
the symptoms, and to ascribe them to a
morbid state of the brain itself, arising,
in all probability, from an aneurismatic
affection of those vessels of the dura mater
which, by passing through the cranium,
communicate with the superficial arteries
of the head, forming, in fact, internally,
a part of the disease which, ivas so con-
spicuous outwardly, and thus by their en-
largement exerting a powerful compres-
sion on the brain itself. This opinion ivas
highly corroborated by symptoms after-
wards noticed, viz. by the great protru-
sion of the eyeball, ancl by the circumstance
that the thrilling pulsation was most ob-
servable in the centre of the tumour, at
that point ivhere the cranium appeared
almost completely absorbed, and where,
ill all probability, the freest communica-
tion existed with the vessels supplying
the membranes of the brain."*-?Lancet,
6th October, 1827.
As there is reason to believe that Mr.
Lawrence is not always obliged to a friend
for a sight of the Lancet, (as he is for
our Journal, of the existence of which he
seems to have accidentally heard some
time in February, 1828!) we ask him
how it happens that he has permitted
such " ERRONEOUS ASSERTIONS" to l'e-
main uncontradicted in the said Lancet,
for more than four months, reserving all
the lire of his indignation for us, who
only re-published the statement?* How
is this long silence of Mr. Lawrence to
be accounted for? Is it possible that,
while poor Nowlan was alive, those in-
genious physiological and pathological
explanations, which we have quoted from
Panton Square, were permitted to glide
down the stream of time, on the pages of
the "Invaluable," as coruscations from
the mighty intellects of Messrs. Lawrence
and Wardrop; but that, four months
afterwards, when poor Nowlan's death
and dissection dissolved, into thin air,
these fairy fabricks of the imagination,
Mr. Lawrence started, all at once, from his
halcyon slumbers?loudly declared that
he had never enunciated any thing of
the kind?that the whole was an " ab-
surdity"?and an " erroneous assertion"
by Dr. Johnson !!
Whether Mr. Lawrence may chance to
" catch a tartar" in this crusade, remains
to be seen. At all events, he has placed
upon record, (inadvertently, no doubt,)
his own SOLEMN TESTIMONY to the MEN-
DACITY of the Lancet :?and, in aiming
a dagger at the veracity of Dr. Johnson,
he has plunged that instrument into the
bosom of his faithful ally?the Lancet!
We are not among those who see the fin-
ger of Providence in every instance of
moral retribution, in this world ; but we
will say, that Mr. Lawrence has brought
about a piece of " dramatic justice," in
this scene, which well deserves to be re-
corded.
As to Mr. Lawrence's strictures on our
taste and feeling?this is the " Devil re-
proving Sin,'" with a vengeance, after the
taste and feeling displayed in " Paul's
Epistles" to his bosom friends?Messrs.
Cooper, Travers, and Butter! We leave
it to the members of the Medico-Chirur-
gical Society, who heard Mr. Lawrence's
statements, to decide on the correctness
or incorrectuess of our report, with just as
much confidence as Mr. Lawrence does.'f
* Sec our last Fasciculus for the beau-
tiful verification of these views.
* The account of this case is acknow-
ledged to be taken from the Lancet, so
that Mr. Lawrence can have no pretence
for the attempt to father it on us.
?f- The statement in the Lancet of last
week, on this subject, looks a little sus-
picious of breach of confidence some-
1828]
Prosecution of Mr. Stanley. 527
We have given insertion to Mr. Law-
rence's letter verbatim, becauseit is short,
and touches on a subject of great intesest
in the surgical world?we mean the late
operation of Mr. Wardrop. We have, in
this, violated a rule which we laid down
at the beginning of the year, and given
just cause of offence to other correspon-
dents. We have treated Mr. Lawrence
with more respect than he can reasonably
claim from us, after the supercilious pre-
amble of his letter j but we shall take
care, in future, not to tax our readers
with altercations between two individuals,
for neither of which, perhaps, the public
care a straw. The Lancet (which now is
absolutely destitute of professional infor-
mation) is the proper channel for this
species of medical literature, and to that
publication we shall refer all applicants.
Prosecution of Mr. Stanley.
It is a very general law, we believe,
among animals, that individuals of the
same species avoid preying on each other.
It is true, that many of them are very
pugnacious. Cocks will fight while life
remains?dogs will worry each other?
and bulls will meet with tremendous clash,
when enfuriated by animal passions. The
history of the human race, unfortunately,
proves that nations will unite to exter-
minate nations, while individuals are per-
petually engaged in the ignoble pursuit
of supplanting, cheating, scandalizing,
and betraying one another! AVith all
these imperfections in poor weak mortals,
there has long existed among the liberal
and learned professions, a kind of uni-
versal compact that men of the same
faculty should obey the Christian pre-
cept?" do unto others as ye would be
done by"?put the most merciful con
struction on things?and never attempt
to exaggerate their neighbour's failings,
or exasperate the passions of the popu-
lace against one of their own brethren.
This tacit compact has very generally
been exemplified in the three learned
professions. The recent action, in which
Mr. Stanley was amerced in 30 pounds
damages, for not discovering the precise
nature of an accident, after swelling and
inflammation of the parts had come on?
and for mistaking a foreign body jammed
along-side or beneath the patella, for
a portion of that bone broken off, has
called forth such a stormy effusion of
vituperation from a junto of his bre-
thren, that human nature sickens at
the scene! Heaven knows we owe Mr.
Stanley no obligation ; nor are we the .
toad-eaters of the Medical Aristocracy
to which he is said to belong ; but were
he our bitterest enemy, we should detest
our own existence, if, for one moment,
we permitted personal feeling to inter-
fere with the dictates of justice. Air.
Stanley may have committed an error in
judgment?errare est humanum?but we
do most solemnly protest against the fiat
of that professional tribunal (if it deserves
such a name) which condemns Mr. Stan-
ley, without knowing all the particulars
of the case, and merely because an igno-
rant jury gave a verdict in direct oppo-
sition to the judge, and to several of the
most eminent surgeons in London. We
have no terms expressive enough of our
indignation, at seeing a portion of the
medical press take advantage of such a
verdict, in order to harrass an individual,
who happens to be one of a different party.
But Mr. Stanley may console himself with
the reflection?nay, with the certainty,
that this procedure of the press, will defeat
its own illiberal purposes?and that the
where?we do not mean on Mr. Law-
rence's part. However, we have but one
short reply to make:?We defy any mem-
ber of the Medico-Chirurgical Society to
shew that we have ever published " mis-
statements," (the term applied in the
Lancet) while reporting the proceedings
of that body. Whoever asserts, by letter,
by word, or by print, that we have mis-
stated, or misrepresented, any case read,
or any opinion given, in that Society,
commits himself a " mis-statement." Qui
capit ille facit.
* If the writers of these inflammatory
effusions were acquainted with the pre-
judices, which medical men have to
encounter in their intercourse with the
public, they would not be so ready, on
all occasions, to increase that prejudice,
by holding up the most eminent men in
the Profession to ridicule. By this pro-
cedure, they put arguments into the
mouths of every little tradesman to scoff
528 Periscope: or, Circumspective Review. [Feb. 23
Profession, so far from joining in the vulgar
outcry, will support him through the
momentary struggle. We have said be-
fore, that we owe Mr. Stanley 110 obliga-
tion, but we owe our tribute to justice,
as well as to generosity?and while un-
merciful critics point their black artillery
at his head, we will publickly and pri-
vately espouse his cause?and so we are
convinced, will every member of the
profession, who has a spark of Chris-
tianity, philanthropy, or magnanimity in
his breast!
In taking this line, we enter not, at all,
into the surgical merits of the case. We
have no proper data on which to ground
a decision?and, till some circumstantial
details of the accident, and the treatment,
be laid before the Profession, we will not
be so uncharitable as to pass a judgment,
much less a censure on the practitioners
in attendance.
Libel?Macleod versus Wakley.
This cause was determined on Monday
Last, in the Court of King's Bench, be-
fore Lord Tenterden. The Court was
crowded to excess. Sir James Scarlet
stated the case to the Jury with great
force. The impression on the audience
was exceedingly strong, from the intrinsic
evidence that nothing was exaggerated?
no fact distorted?no expression in the
libel coloured. Sir James wound up his
at, and insult the general practitioner
in attendance, whenever he claims a re-
ward for his services. This we see done
every day?and this, every practitioner
is aware of; but the literary garreteer,
who fires oft' his inflammatory harangues
among the public, knows and feels nothing
of the matter.
The Lancet boasts of having been the
instrument that enlightened the Jury to
give damages against Mr. Stanley, in this
case, contrary to the opinion of the Judge,
and against the evidence of a Cooper, a
Brodie, a Bell, a Travels, &c. &c ! Be
it so ! If the Lancet has been able to
raise this prejudice in the minds of the
" profanum vulgus," against such men, it
requires but little divination to see that,
henceforth, no medical practitioner is
one hour safe from prosecution! " These
are thy glorious fruits?parent of ill!"
charge, by reading, from the Lancet of
last Saturday, the prediction, that " the
yellow Goth would be scarified by Mr.
Brougham on Monday." This passage
excited strong symptoms of disgust in
the countenances of judge, jury, and au-
ditors. Mr. Brougham felt it necessary
to apologize for the bad taste of his client,
and moved heaven and earth to keep this
passage from the face of the record. At
length he succeeded by a legal quibble?
namely, a denial that there was any proof
of Mr. Wakley being editor of the Lancet
during the last week ! In this he suc-
ceeded. But the moral efl'ect of the pas-
sage was produced beyond redemption.
Mr. Brougham did all that man could
do?(but, under the circumstances in
which he was placed, his exertions were
of little avail)?to persuade the jury, that
the false assertions against Dr. Macleod
originated in a mistake, and that the vio-
lent language in which they svere con-
veyed, was a mere ebullition of feeling for
an injured friend?Mr. Wardrop. But,
in this delicate position, Mr. Brougham
shewed admirable tact, sound sense, and
consummate policy. Instead of comply-
ing with the savage anticipation of his
client, '' the scarification of the yellow
Goth," not a single expression was per-
mitted to escape his lips, which could
hurt the feelings of the most sensitive
mind on earth! On the contrary, he paid
Dr. Macleod many handsome compli-
ments, in lieu of the expected scarifica-
tions ! By this judicious procedure, and
by the non attemptof the plaintiff to prove
any damages, the defendant was probably
saved from a heavy amercement. The
verdict was all that could be expected,
and five pounds were awarded,which carry
the costs of both sides.
The charge of Lord Tenterden (Judge
Abbott) will long be remembered by the
defendant. Not a single palliating cir-
cumstance was mentioned to the jury.
The accusations (his Lordship observed)
against Dr. Macleod were proved to be
unfounded in truth?and if the jury were
not convinced of this, they ought to find
for the defendant! We suppose a full
account of this trial will be published.
Never were judge, jury, and knights of
the long robe, so puzzled with carotids,
ligatures citra et ultra tumores, post-
mortem researches after vanished aneu-
risms, &c. as on this occasion.

				

